# *In Vivo* pharmacokinetic interactions of ribociclib with rivaroxaban and apixaban in rats: implications for increased drug exposure and dose adjustments

**DOI:** 10.3389/fphar.2025.1530806

**Published:** 2025-03-31

**Authors:** Zihan Liu, Wenyu Du, Qimin Wang, Zhi Wang, Jing An, Yinling Ma, Zhanjun Dong, Ying Li

**Affiliations:** ^1^ Graduate School, Hebei Medical University, Shijiazhuang, China; ^2^ Department of Pharmacy, Hebei General Hospital, Hebei Key Laboratory of Clinical Pharmacy, Shijiazhuang, China

**Keywords:** cancer-associated venous thromboembolism, drug-drug interaction, ribociclib, apixaban, rivaroxaban

## Abstract

**Background:**

Apixaban (API) and rivaroxaban (RIVA) are orally available inhibitors of coagulation factor Xa and are commonly used to treat cancer-related venous thrombosis. Ribociclib (RIBO), a first-line treatment for hormone receptor-positive/human epidermal growth factor receptor 2 negative (HR^+^/HER2^−^) advanced breast cancer, is an inhibitor of CYP3A4, P-gp, and BCRP. Given the potential for these drugs to be co-administered in clinical settings, there is limited information regarding the pharmacokinetic drug-drug interactions (DDIs) between ribociclib and these anticoagulants. This study aimed to evaluate the extent of DDIs between ribociclib and rivaroxaban or apixaban in rats and to explore the optimization of drug dosing strategies.

**Methods:**

Male Sprague-Dawley rats were divided into 9 groups (n = 6), receiving ribociclib, apixaban, rivaroxaban, ribociclib with rivaroxaban, ribociclib with apixaban, and combinations with reduced doses and time intervals. Blood concentrations were measured using ultra-performance liquid chromatography-tandem mass spectrometry (UPLC-MS/MS). Pharmacokinetic parameters such as AUC, C_max_, CL_z_/F, and V_z_/F.

**Results:**

Ribociclib significantly increased exposure to both rivaroxaban and apixaban, with a greater impact on rivaroxaban. Specifically, ribociclib increased the AUC_0-t_, AUC_0- ∞_ and C_max_ of rivaroxaban (normal dose) by about 2.4-fold, 2.1-fold and 1.8-fold, while increasing apixaban exposure by about 60.82%, with a trend towards an increase in C_max_ that was not statistically significant. When co-administered with ribociclib, even at a reduced dosage of 1 mg/kg, rivaroxaban exhibited a significant increase in exposure, with the AUC increasing by 2.3-fold and C_max_ by 1.3-fold. Despite the reduction in dosage, the pharmacokinetic effect of ribociclib on rivaroxaban persisted. While administration of rivaroxaban 12 h after ribociclib resulted in a less pronounced increase in exposure compared to the normal-dose group. The results of qRT-PCR showed that ribociclib reduced the expression of Cyp3a1 and Abcg2 in rat intestine.

**Discussion:**

This research highlights the need for careful consideration of dosing regimens to minimize toxicity risk and optimize the safety of clinical co-administration of ribociclib with rivaroxaban.

## 1 Introduction

Venous thromboembolism (VTE) is the most common complication of cancer, with approximately 15% of patients with cancer experiencing at least one cancer-associated thrombosis (CAT). Conventional cancer treatments often exacerbate the pre-CAT state (Amin et al., 2024; Dickson et al., 2022; Chen, 2024). While CAT is relatively rare in breast cancer compared to other solid cancers, breast cancer remains the most common cancer among women worldwide, and the number of breast cancer-associated thrombosis (BrCAT) is significantly high (Bray et al., 2024). The treatment of CAT is associated with a higher incidence of thrombotic recurrence and/or major bleeding compared to non-cancer VTE (Fernandes et al., 2019; Riaz et al., 2023). According to clinical guidelines, low molecular heparin (LMWH) has historically been the preferred regimen for the treatment of CAT (Kahn et al., 2012; Key et al., 2020; Mandala et al., 2011). However, the necessity and high cost of daily subcutaneous injections have reduced patient compliance (Wittkowsky, 2006). Recent randomized clinical trials have demonstrated that oral direct anticoagulants (DOACs) are as effective as LMWH in the acute management of CAT, with a lower rate of VTE recurrence (Agnelli et al., 2020; Schrag et al., 2023; Marcucci et al., 2022; Amin et al., 2024). DOACs also offer practical advantages, such as not requiring frequent monitoring of the International Normalized Ratio (INR), which enhances ease of use and cost-effectiveness for patients. As a result, international clinical practice guidelines have increasingly supported the use of DOACs as an alternative to LMWH monotherapy for both the initial and long-term treatment of CAT. Despite their benefits, the adoption of DOACs necessitates careful consideration due to certain limitations. Notably, DOACs are associated with a higher risk of bleeding and potential drug-drug interactions (DDIs) with some anticancer therapies (Frere et al., 2022; Lyman et al., 2021).

Apixaban is a small-molecule, selective factor Xa inhibitor that inhibits both free and clot-bound factor Xa and has been approved for the clinical treatment of several thromboembolic diseases, including the prevention of VTE (Jiang et al., 2009). Apixaban is primarily metabolised by the cytochrome P450 (CYP) enzyme CYP3A4/5, with lesser involvement of CYP1A2, CYP2C8, CYP2C9, CYP2C19, and CYP2J2 (Byon et al., 2019). Additionally, it is a substrate for P-glycoprotein (P-gp) and breast cancer resistance protein (BCRP) (Zhang et al., 2013). Rivaroxaban is another direct factor Xa inhibitor, with a higher selectivity for this coagulation factor. It is rapidly absorbed, reaching maximum plasma concentration (C_max_) within 2–4 h after administration (Kvasnicka et al., 2017). Its pharmacokinetics and pharmacodynamics are proportional to the dose. Rivaroxaban is metabolized by CYP3A4/5 and CYP2J2, with CYP3A4/5 accounting for approximately 18% of the total elimination and CYP2J2 for approximately 14% (Mueck et al., 2013). Additionally, non-CYP-dependent pathways also contribute to the elimination of rivaroxaban. Combinations therapies can lead to DDIs that affect the exposure or pharmacological activity of DOACs. Both potent inhibitors and inducers of P-gp or CYP3A4 are known to significantly influence DOAC pharmacokinetics and efficacy (Bellesoeur et al., 2018).

The efficacy of cyclin-dependent kinase (CDK) 4/6 inhibitors in treating breast cancer is well established, making them the first-line therapy for hormone receptor-positive/human epidermal growth factor receptor 2 negative (HR^+^/HER2^−^) breast cancer (Pavlovic et al., 2023; Mayer et al., 2021; Lee et al., 2023). Among these, ribociclib is a novel CDK4/6 inhibitor that has shown efficacy in the treatment of advanced breast cancer and was approved by the Food and Drug Administration (FDA) in 2017. The recommended starting dose of ribociclib is 600 mg once daily (Braal et al., 2021). Ribociclib is mainly metabolized by CYP3A4 and is a substrate for P-gp. At the dose of 600 mg, ribociclib is a potent inhibitor of CYP3A4, and significantly inhibits P-gp and BCRP (Sorf et al., 2018; Kulkarni and Singh, 2024).

Co-administration of ribociclib with midazolam, a sensitive CYP3A4 substrate, has been shown to increase midazolam exposure (Samant et al., 2020). Consequently, we postulate that the co-administration ribociclib with DOACs may alter the exposure or pharmacological activity of DOACs. However, there is currently a paucity of data on the DDIs between ribociclib and DOACs, such as rivaroxaban and apixaban. Such DDIs could increase the risk of haemorrhage recurrence or recurrent VTE (Sabatino et al., 2020). Furthermore, DDIs may decrease the efficacy and safety of anticancer treatments or other medications employed for managing comorbidities. At present, clinical evidence on ribociclib-DOAC interactions is currently limited, particularly in patients with cancer. It is therefore imperative that these interactions are comprehensively studied to ensure the safe co-administration of these drugs and inform dosage adjustments (Tsoukalas et al., 2022).

The objective of the present study was to examine the potential DDIs between ribociclib and rivaroxaban or apixaban. To this end, the study comprised several key steps aimed at evaluating the pharmacokinetic interactions between ribociclib, apixaban and rivaroxaban Rats were assigned to different treatment groups and administered the three drugs either as monotherapy or in combination with ribocicliband apixaban or rivaroxaban. Ultra-performance liquid chromatography-tandem mass spectrometry (UPLC-MS/MS) was employed to detect the blood drug concentrations at different time points. Pharmacokinetic parameters were then calculated and observed drug interactions were subjected to statistical analysis. All materials, experimental procedures, and validation methods were rigorously designed and executed to ensure the accuracy, reliability, and reproducibility of the data obtained.

## 2 Materials and methods

### 2.1 Study design and treatments

#### 2.1.1 Animals

Adult specific pathogen-free (SPF)-grade male Sprague-Dawley (SD) rats weighing 230 ± 30 g were provided by Beijing Huafukang Biotechnology Co., LTD., (Beijing, China; license number SCXK (Jing) 2019-0008). The study adhered to the ARRIVE guidelines (Percie et al., 2020), and all mouse procedures were conducted under humane process. The study was approved and supervised by the Animal Ethics Committee of Hebei General Hospital (Shijiazhuang, China) (No.2024DW-072).

Before the experiment, the rats were acclimated to standard laboratory conditions, including a 12-h light/dark cycle, a temperature of 23 ± 2°C, and a relative humidity of 50% ± 10%. They were provided with adequate food and water during a one-week adaptive feeding period. Food was withheld for 12 h before drug administration to ensure standardized conditions for the experiment.

#### 2.1.2 Pharmacokinetic study in rats

Experimental animals were randomly divided into nine groups (n = 6, Table 1). Ribociclib was suspended in methylcellulose (MC), rivaroxaban was prepared in hydroxypropyl methylcellulose (HPMC), and apixaban was suspended in 5% DMSO in water. To achieve steady-state blood concentrations, a drug typically requires five to seven half-lives. Based on this principle, ribociclib was administered orally for eight consecutive days, while rivaroxaban and apixaban were administered orally for five consecutive days to ensure steady-state concentrations were reached before further analysis.

**TABLE 1:** Study design.

Study design	Group No.	Treatment description
The influence of RIBO on RIVA pharmacokinetics		
RIVA 2 mg/kg alone	Group I	RIVA 2 mg/kg alone
RIBO + RIVA	Group II	RIBO 60 mg/kg for 8 consecutive days + RIVA 2 mg/kg
RIBO + RIVA (12 h prior)	Group III	RIBO 60 mg/kg for 8 consecutive days + RIVA 2 mg/kg 12 h before the dose
RIBO + Reduced RIVA	Group IV	RIBO 60 mg/kg for 8 consecutive days + RIVA 1 mg/kg
The Influence of RIBO on API Pharmacokinetics		
API 0.5 mg/kg alone	Group V	API 0.5 mg/kg alone
RIBO + API	Group Ⅵ	RIBO 60 mg/kg for 8 consecutive days + API 0.5 mg/kg
The Influence of RIVA or API on RIBO Pharmacokinetics		
RIBO 60 mg/kg alone	Group Ⅶ	RIBO 60 mg/kg alone
API + RIBO	Group Ⅷ	API 0.5 mg/kg for 5 consecutive days + RIBO 60 mg/kg
RIVA + RIBO	Group Ⅸ	RIVA 2 mg/kg for 5 consecutive days + RIBO 60 mg/kg

#### 2.1.3 Blood sample

Approximately 0.1 mL of blood was collected into heparinized tubes via the orbitalat specified time points before and after the final drug administration. For apixaban, blood samples were collected at 0, 0.083, 0.167, 0.25, 0.333, 0.5, 0.75, 1, 3, 5, 7, 10, and 12 h. For rivaroxaban, sampling was conducted at 0, 0.167, 0.333, 0.5, 0.75, 1.5, 2, 3, 4, 5, 6, 8, 10, 12, and 24 h. For ribociclib, samples were taken at 0, 0.5, 1, 2, 4, 5, 6, 8, 10, 12, 24, and 48 h. Blood samples were centrifuged at 3,500 rpm for 10 min, and the supernatant was collected and stored at −80°C until processing for UPLC-MS/MS.

### 2.2 UPLC-MS/MS assay

#### 2.2.1 Chemicals and reagents

Ribociclib (purity>98%, Lot EMC104) was supplied by Bido Pharmaceutical Co., Ltd. Ribociclib-d_6_ (purity>99%, Lot IR-67675) and apixaban (purity ≥98%, Lot C15069980) were purchased from Shanghai Macklin Bio-Technology Co. Ltd., (Shanghai, China). Rivaroxaban (purity ≥99%, Lot H25J9Z64216) was provided by Shanghai yuan ye Bio-Technology Co. Ltd., (Shanghai, China). Rivaroxaban-d_4_ (purity >98%, Lot 21,702) was obtained from B1203 Life Science Park, SCT Creative Factory. (Shenzhen, China). Dimethyl sulfoxide (DMSO) was acquired from Beijing Solarbio Science Technology Co. Ltd., (Beijing, China). Acetonitrile, formic acid, ammonium acetate and methyl tert-butyl ether were of high-performance liquid chromatography (HPLC) grade and supplied by Fisher Scientific (Pittsburgh, PA, United States). Ultrapure water was obtained from Wahaha Group Co., Ltd., (Hangzhou, China).

#### 2.2.2 Instrumentation and chromatographic conditions

Apixaban and rivaroxaban concentrations in plasma samples were determined using our previously established UPLC-MS/MS method (Wang et al., 2023). Ribociclib concentrations were quantified using an LC-30A UPLC system (Shimadzu, Kyoto, Japan) coupled with a Sciex Triple Quad 5500 tandem quadrupole mass spectrometer (AB Sciex, Framingham, MA, United States) equipped with an electrospray ionization interface. Chromatographic separation was performed using a Welch Boltimate column (2.1 mm × 100 mm, 2.7 μm) maintained at 40°C. The mobile phase was composed of water containing 0.1% formic acid (phase A) and acetonitrile (phase B). The flow rate was held constant at 0.3 mL/min. The gradient elution protocol was as follows: 0–2.5 min, 60% B; 2.5–3.5 min, 60%–90% B; 3.5–5.5 min, 90% B; 5.5–5.6 min, 90%–60% B; 5.6–6.6 min, 60% B. The injection volume was 1 μL.

**FIGURE 1:**
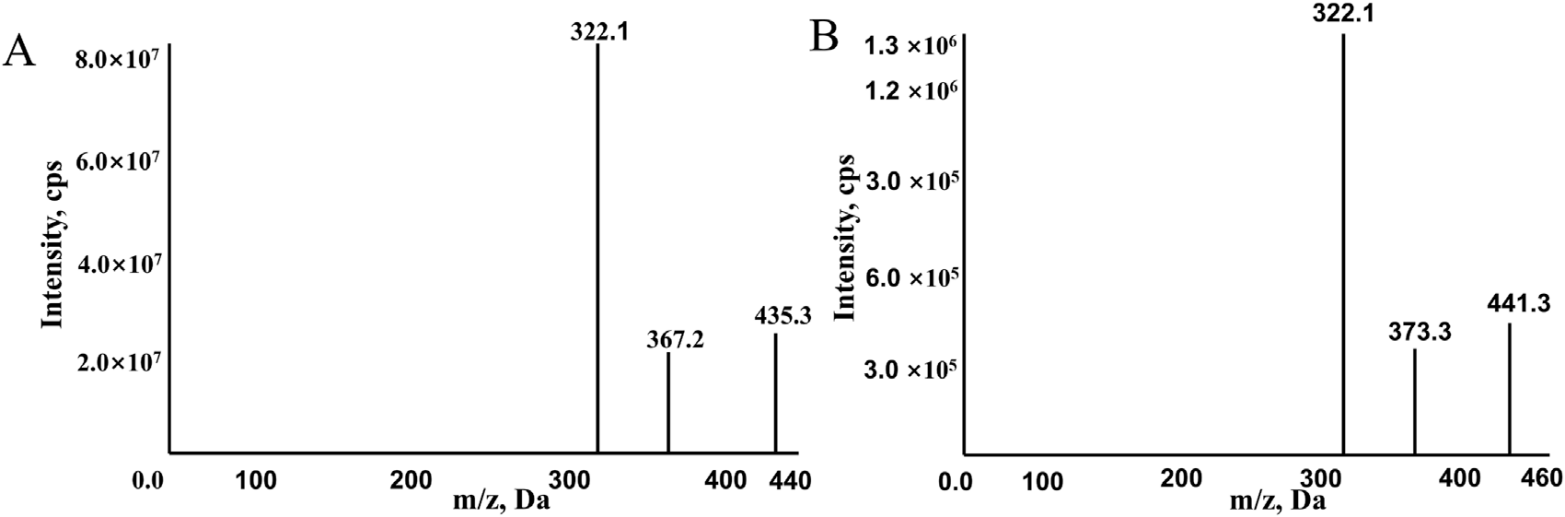
The mass spectra of RIBO **(A)** and RIBO-d_6_ **(B)**.

The mass spectrometer operated in positive ion mode and multiple reaction monitoring (MRM) mode for detection and quantification. The monitored transitions of precursors to product ions were as follows: 441.3→332.1 for ribociclib-d_6_ and 435.3.→322.1 for ribociclib (Figure 1). The mass spectrometer conditions, including delustering potential (DP) and collision energy (CE) of the compounds, are shown in Table 2. Other parameters of the mass spectrometer were as follows: ion source gas 160.0 psi; ion source gas 2,50.0 psi; curtain gas, 25.0 psi. The source temperature was 500°C and the ion spray voltage was 5,500 V.

**TABLE 2:** The experimental setting for the tandem mass-spectrometer for the analytes and internal standards.

Experimental setting	RIBO	RIBO-d_6_
MRM transition	435.3→322.1	441.3→332.1
Delustering potential (DP), V	130	130
Collision energy (CE), V	35	35
Collision cell exit potential (CXP), V	14	14
Entrance potential (EP), V	10	10

#### 2.2.3 Preparation of calibration standards and quality control (QC) samples

Stock solutions of 1-mg/mL ribociclib and ribociclib-d_6_ (IS) were prepared separately in DMSO. Working solutions were prepared by diluting the standard stock solutions with 50% (v/v) acetonitrile in water. Calibration standards were prepared by spiking 5 μL of the working solution into 45 μL of blank rat plasma. The stock solutions were further diluted with 50% (v/v) acetonitrile in water to create working solutions of varying concentrations. The final concentrations for calibration curves were as follows: 10, 50, 100, 200, 400, 800, 2,000, and 4,000 ng/mL of ribociclib. Low-, medium-, and high-concentration QC samples were processed independently and contained 20, 500, and 3,000 ng/mL.

#### 2.2.4 Method validation

The method was validated per the guidelines of the Food and Drug Administration (US-FDA, 2022) and Chinese Pharmacopoeia (2020). Selectivity was determined by analyzing blank plasma samples from six different batches of rats and plasma samples from six different batches of rats after administration of ribociclib. There should be no interference, with retention times such that the blank plasma peak area is less than 5% of the internal standard (IS) and less than 20% of the lower limit of quantification (LLOQ). The calibration curves were evaluated using 10–4,000 ng/mL of ribociclib. Linearity was assessed by plotting the peak area ratios of the analyte to the IS against nominal concentrations, using weighted (1/x^2^) least-squares linear regression. Accuracy and precision were determined for all concentrations derived from the calibration curves, ensuring that deviations were within 15% of the nominal concentration, except for the LLOQ, where the deviation should not exceed 20%. Precision and accuracy were assessed by analyzing plasma samples at low, medium, high, and LLOQ concentration levels over three consecutive days. The final precision and accuracy were determined by calculating the relative standard deviation (RSD) and relative error (RE) of the six samples. RSD and RE for QC concentrations were required to be within ±15%, and for the LLOQ, within ±20%. Matrix effects were assessed by comparing analyte peak areas in blank plasma samples of low, medium, and high concentration QC samples (n = 6) with analyte peak areas in the corresponding pure solutions. Extraction recovery was assessed by comparing the peak areas of analytes in extracted plasma samples from QC samples at three concentrations (n = 6) with the peak areas of analytes in blank plasma extracts at the same concentrations. Analyte stability was assessed at low, medium, and high QC concentrations under four different storage and handling conditions: room temperature (25°C) for 4 h, autosampler temperature (15°C) for 6 h, −80°C for 30 days, and three freeze-thaw cycles of plasma samples (−80°C–25°C). The RSD of all QC samples should be less than 15%, and the RE should be between 85% and 115% of the labeled concentration.

### 2.3 Quantitative real-time PCR (qRT-PCR) analysis

qRT-PCR analysis was used to determine the mRNA levels of Cyp3a1(CYP3A4) in the liver and Abcb1a (P-gp), Cyp3a1(CYP3A4), Abcg2(BCRP) in the intestines. Total RNA was extracted from frozen liver and intestine samples using the TRNzol Universal reagent, following the manufacturer’s instructions. The Bio Tek Epoch (Bio Tek Instruments, Inc., Winooski, VT, United States) was used to quantify the purity and concentration of the total RNA, based on the ratio of the absorbance at 260 and 280 nm. The RNA samples, each containing 1 µg of material, were converted to complementary DNA (cDNA) using the FastKing RT Kit. The real-time PCR assays were performed using a two-step amplification protocol on the SLAN-96S Real-Time PCR system (Shanghai Hongshi Medical Technology Co., Ltd., Shanghai, China). NADPH was employed as an internal control, and the PCR cycling criteria were as follows: 95°C for 15 min, followed by 40 cycles of 95°C for 10 s and 60°C for 32 s. The sequences of the primers are presented in Table 3.

**TABLE 3:** Primer sequences used in quantitative RT-PCR.

Gene	Forward primer	Reverse primer
Cyp3a1	5′-TGC​ATT​GGC​ATG​AGG​TTT​GC-3′	5′-TTC​AGC​AGA​ACT​CCT​TGA​GGG-3′
Abcb1a	5′-TCT​GGT​ATG​GGA​CTT​CCT​TGG​T-3′	5′-TCC​TTG​TAT​GTT​GTC​GGG​TTT​G-3′
Abcg2	5′-TGA​AGA​GTG​GCT​TTC​TAG​TCC​G-3′	5′-TTG​AAA​TTG​GCA​GGT​TGA​GGT​G-3′
NADPH	5′-GCC​TTC​CGT​GTT​CCT​ACC-3′	5′-GCC​TGC​TTC​ACC​ACC​TTC-3′

### 2.4 Pharmacokinetic analysis

#### 2.4.1 Plasma sample preparation

A 50 µL plasma sample was mixed with 5 µL of the IS working solution, followed by the addition of 150 µL of acetonitrile. The mixture was vortexed for 2 min and then centrifuged at 12,000 rpm for 10 min. Next, 70 µL of the supernatant was collected and mixed with 70 µL of 50% (v/v) of acetonitrile in water. The mixture was vortexed for 1 min, and then 1 µL was injected into the UPLC-MS/MS system for analysis.

#### 2.4.2 Statistical analysis

Pharmacokinetic parameters were calculated based on a non-compartmental model using DAS 2.1.1 Software (Mathematical Pharmacology Professional Committee of China, Shanghai, China). The area under the concentration–time curve (AUC), maximum plasma concentration (C_max_), time to maximum plasma concentration (T_max_), the time required to eliminate half of the plasma drug concentration (t_1/2_), clearance of drug plasma volume per unit time (CL_z_/F), apparent volume distribution (V_z_/F), mean residence time (MRT) and the absorption rate constant (Ka) are all expressed as mean ± standard deviation (SD). SPSS 25.0 statistical software (SPSS Inc., Chicago, IL, United States) was applied to statistically analyze the mainpharmacokinetic parameters. Shapiro-Wilk test was used to assess whether the data were normally distributed, t test was used for parameters conforming to the normal distribution, and Mann-Whitney U test was used for parameters not conforming to the normal distribution. Statistical comparisons were conducted using a analysis of variance, t-test or nonparametric rank-sum test depending on the data type. A P-value <0.05 was deemed statistically significant.

## 3 Results

### 3.1 Method development and optimization

**FIGURE 2:**
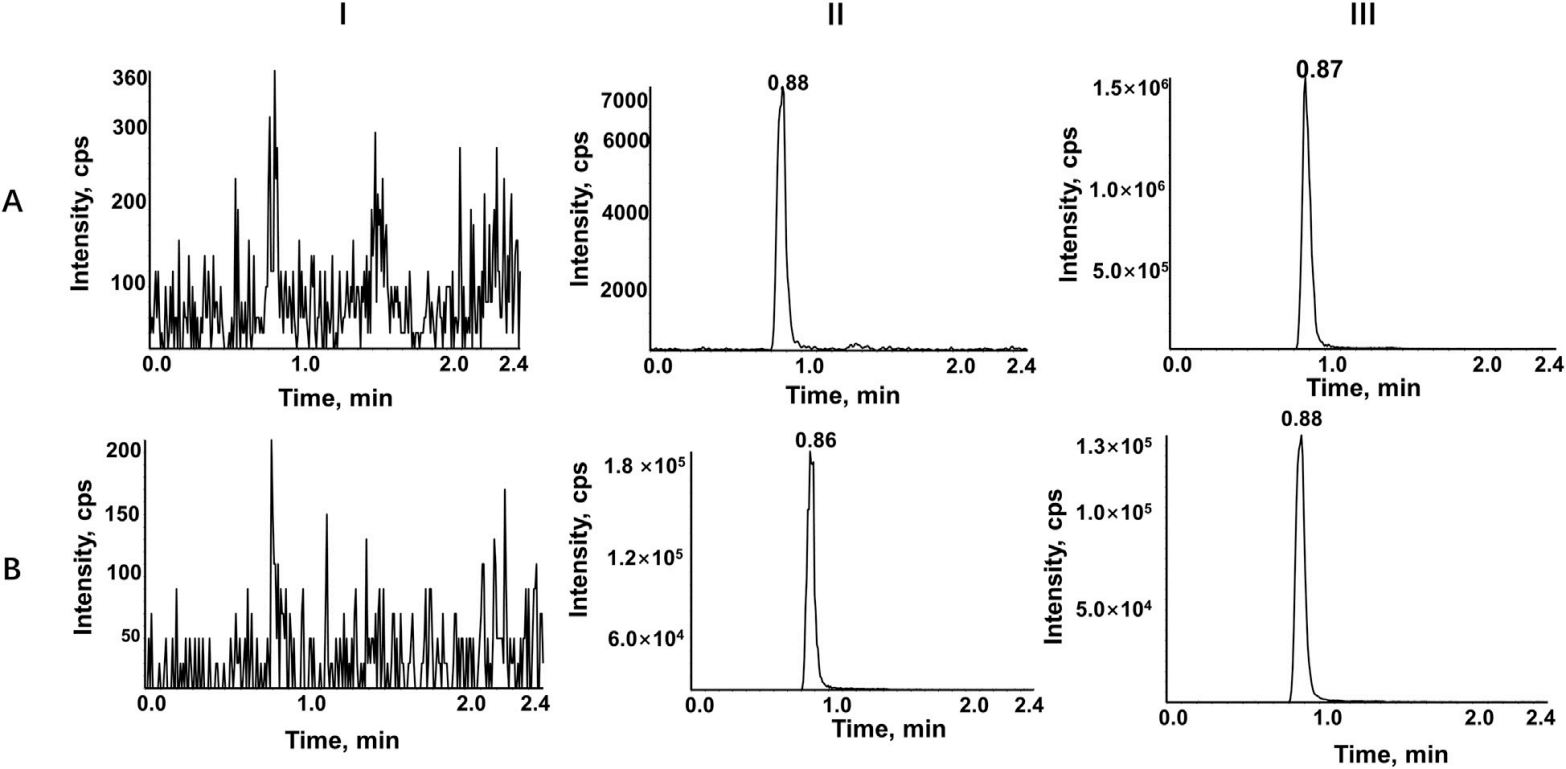
Representative chromatograms of RIBO **(A)**, and RIBO-d_6_ **(B)**. Ⅰ, a blank rat plasma sample; Ⅱ, a blank rat plasma sample spiked with the working solution at LLOQ level and IS; Ⅲ, a rat plasma sample after oral administration of 60 mg/kg RIBO.

A highly sensitive and reproducible UPLC-MS/MS method was developed to evaluate the pharmacokinetic interactions of ribociclib in rats. The chromatographic conditions were optimized to obtain good peak symmetry, high detection sensitivity and a short retention time. Given that acetonitrile exhibits superior elution ability relative to methanol, it was selected as the organic phase, and formic acid was introduced to augment the chromatographic signal and peak spectrum of ribociclib. A gradient elution method was employed, starting with 40% (v/v) acetonitrile at a flow rate of 0.3 mL/min. This approach yielded high detection sensitivity and a short retention time. Ribociclib-d_6_, a deuterated analogue of ribociclib with similar physicochemical properties and identical mass spectral characteristics was selected as the IS to enhance the accuracy and precision of the analysis. Figure 2 shows typical chromatograms of ribociclib and ribociclib-d_6_ in different plasma samples. No significant interference from endogenous substances was detected. Calibration curves were constructed using linear regression analysis across a concentration ranges of 10–4,000 ng/mL for ribociclib. A typical calibration curves was described by the equation Y = 0.00332 X + 0.00874 (r = 0.9987) for ribociclib. Intra- and inter-day precision values were below more than 6.3%, and the accuracies ranged from −7.6% to 10.7% for all investigated analyte concentrations in rat plasma. Matrix effects for ribociclib ranged from 90.1% to 106.5%, indicating no significant matrix effect existed in rat plasma. Recovery rates, normalized by IS peak area, ranged from 97.7% to 108.1%, and the RSD was less than 5.63% for the analyte. Stability tests for QC samples, conducted under various conditions—including room temperature, autosampler storage at 15°C, −80°C, and after three freeze-thaw cycles—demonstrated excellent stability. Relative error (RE) values were below 5.8%, and RSD values were less than 6.7%. These results indicate that the developed method is repeatable and reproducible.

### 3.2 Pharmacokinetic study

#### 3.2.1 Effect of ribociclib on the pharmacokinetics of rivaroxaban

**FIGURE 3:**
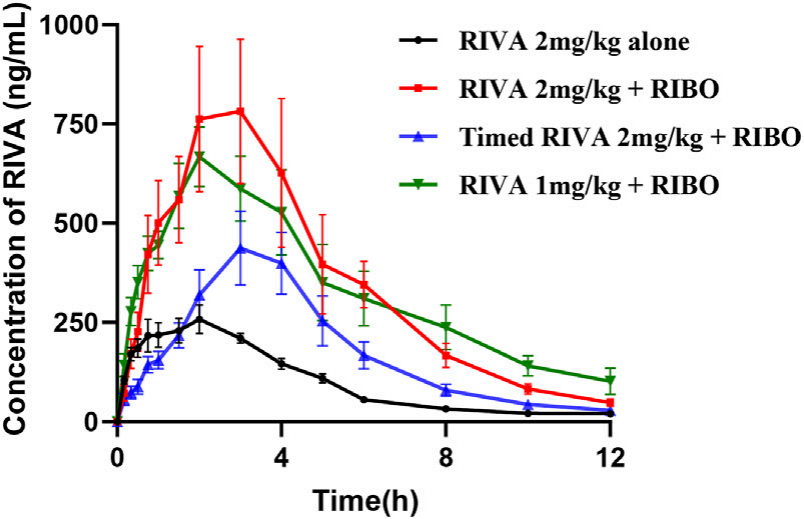
Mean plasma concentration-time profiles of RIVA alone and in combination with multiple doses and timed administration of RIBO.

The mean plasma concentration-time profiles of rivaroxaban alone and in combination with multiple doses and timed administration of ribociclib are shown in Figure 3. The main pharmacokinetic parameters of rivaroxaban are summarized in Table 4. After multiple doses of ribociclib, the AUC_0-t_, AUC_0- ∞ _, and C_max_ of rivaroxaban increased by 240.19% (P = 0.002), 213.79% (P = 0.002), and 186.93% (P = 0.002), respectively, compared to rivaroxaban alone at 2 mg/kg. CL_z_/F, t_1/2z_, and V_z_/F decreased by 65.81% (P < 0.001), 57.09% (P = 0.015), and 82.91% (P = 0.002), respectively. MRT_0-t_ increased by 21.39% (P = 0.04). The differences in the other pharmacokinetic parameters, T_max_ and MRT_0- ∞ _, were not statistically significant. Compared to rivaroxaban 2 mg/kg, rivaroxaban AUC_0-t_ and AUC_0- ∞_ increased by 74.16% (P = 0.023) and 63.03% (P = 0.035), respectively, and CL_z_/F and V_z_/F decreased by 34.84% (P = 0.033) and 62.61% (P = 0.004), respectively, with multiple doses of rivaroxaban followed by a 12-h interval of ribociclib. MRT_0-t_ increased by 22.54% (P = 0.041). The differences in the other pharmacokinetic parameters T_max_, C_max_, CL_z_/F, and MRT_0- ∞_ were not statistically significant. Ribociclib co-administration with rivaroxaban 1 mg/kg in multiple doses also led to increases in Compared to rivaroxaban 2 mg/kg, multiple dosing of ribociclib increased AUC_0-t_, AUC_0- ∞ _, and C_max_ of rivaroxaban by 230.62% (P < 0.001), 228.46% (P < 0.001), and 133.14% (P = 0.002), respectively, compared to the administration of rivaroxaban alone at 2 mg/kg. Furthermore, CL_z_/F and V_z_/F decreased by 70.97% (P < 0.001) and 78.29% (P < 0.001), respectively. MRT_0-t_ increased by 30.06% (P < 0.001), while the differences T_max_, CL_z_/F, and MRT_0- ∞_ were not statistically significant.

**TABLE 4:** Pharmacokinetic parameters of RIVA in rats when administered alone and following simultaneous and timed administration of RIBO.

Parameters (Unit)	RIVA 2 mg/kg			RIVA 1 mg/kg + RIBO
	Alone	+ RIBO	+ Timed RIBO (12 h)	
AUC_0-t_ (μg/L*h)	1,206.06 ± 197.70	4,102.87 ± 2003.47**	2,100.44 ± 794.79*	3987.53 ± 444.98**
AUC_0- ∞ _(μg/L*h)	1,359.33 ± 325.65	4,267.49 ± 1959.63**	2,217.15 ± 800.81*	4,467.05 ± 546.91**
C_max_ (μg/L)	292.17 ± 67.51	838.33 ± 480.92**	454.5 ± 214.88	681.17 ± 75.53*
T_max_ (h)	2.13 ± 0.83	2.83 ± 0.41	3 ± 0.63	1.92 ± 0.20
t_1/2_ (h)	5.01 ± 3.30	2.15 ± 0.94*	2.65 ± 1.48	3.34 ± 0.79
CL_z_/F (L/h/kg)	1.55 ± 0.39	0.53 ± 0.16**	1.01 ± 0.37*	0.45 ± 0.06**
V_z_/F (L/kg)	9.95 ± 4.58	1.70 ± 0.99**	3.72 ± 1.98**	2.16 ± 0.04*
MRT_0-t_ (h)	3.46 ± 0.43	4.20 ± 0.63*	4.24 ± 0.69*	4.50 ± 0.25**
MRT_0- ∞ _(h)	5.31 ± 2.01	4.74 ± 0.63	4.93 ± 1.30	5.84 ± 0.81

*P < 0.05, **P < 0.01, compared to RIVA, alone. Pharmacokinetic parameters are expressed as mean ± standard deviation.

#### 3.2.2 Effect of ribociclib on the pharmacokinetics of apixaban

**FIGURE 4:**
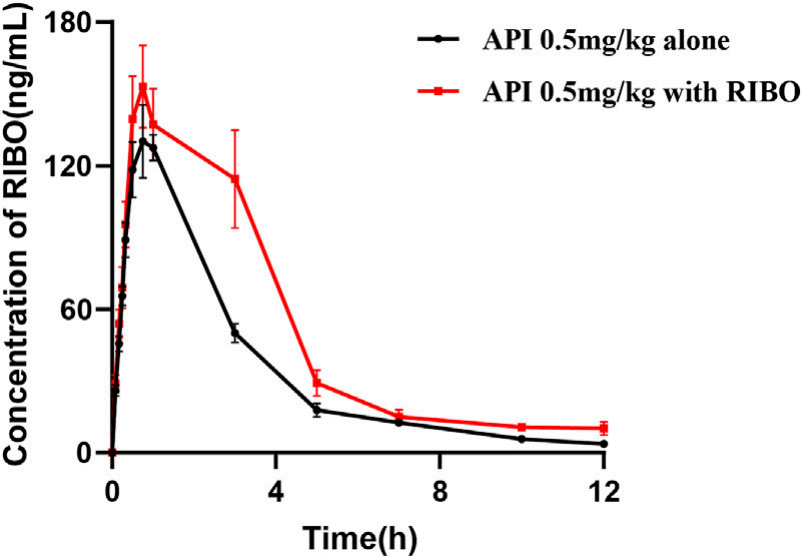
The mean plasma concentration-time graphs of API after oral administration alone and following multiple doses of RIBO.

Figure 4 presents the mean plasma concentration-time curves of apixaban (0.5 mg/kg) administered alone or concomitantly with ribociclib, while Table 5 summarizes the main pharmacokinetic parameters of apixaban. After multiple administrations of ribociclib, the AUC0- ∞ , t_1/2z_, MRT_0-t_, and MRT_0- ∞_ of apixaban were increased by 60.82% (P = 0.026), 115.90% (P = 0.015), 14.45% (P = 0.016), and 61.41% (P = 0.004), respectively, compared to treatment with apixaban-alone. CL_z_/F was increased by 32.23% (P = 0.026). The differences in other pharmacokinetic parameters, including AUC_0-t_, T_max_, CL_z_/F and C_max_ were not statistically significant.

**TABLE 5:** Pharmacokinetic parameters of API in rats after oral administration alone and following multiple doses of RIBO.

Parameters (Unit)	API 0.5 mg/kg	
	Alone	After RIBO Donafenib oddonaDAPA
AUC_0-t_ (μg/L*h)	408.82 ± 55.29	608.21 ± 196.17
AUC_0- ∞ _ (μg/L*h)	419.57 ± 54.24	674.76 ± 221.39*
C_max_ (μg/L)	140.17 ± 29.44	156.5 ± 42.44
T_max_ (h)	0.88 ± 0.14	0.75 ± 0.16
t_1/2_ (h)	2.39 ± 0.53	5.16 ± 3.22*
CL_z_/F (L/h/kg)	1.21 ± 0.14	0.82 ± 0.30*
V_z_/F (L/kg)	4.16 ± 1.07	5.57 ± 2.63
MRT_0-t_ (h)	2.63 ± 0.10	3.01 ± 0.30*
MRT_0- ∞ _(h)	2.98 ± 0.32	4.81 ± 2.07**

*P < 0.05, **P < 0.01, compared to API, alone. Pharmacokinetic parameters are expressed as mean ± standard deviation.

#### 3.2.3 Effect of rivaroxaban or apixaban on the pharmacokinetics of ribociclib

**FIGURE 5:**
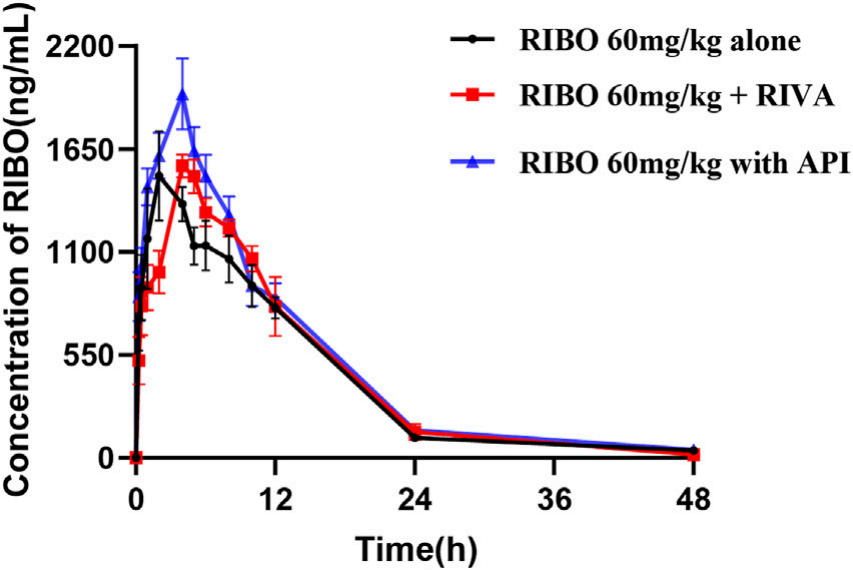
Mean plasma concentration-time profiles of RIBO after oral administration alone and following multiple doses of RIVA or API.

Figure 5 shows the plasma concentration-time curves of ribociclib after administration of ribociclib alone and in combination with multiple doses of rivaroxaban or apixaban, and the pharmacokinetic parameters are shown in Table 6. There were no significant changes in the pharmacokinetic parameters of ribociclib after co-administration with rivaroxaban or apixaban.

**TABLE 6:** Pharmacokinetic parameters of RIBO in rats after oral administration alone and following multiple doses of RIVA or API.

Parameters (Unit)	RIBO (60 mg/kg)		
	Alone	After RIVA Donafenib oddonaDAPA	After API
AUC_0-t_ (μg/L*h)	20,283.32 ± 4,314.32	21,367.71 ± 2,809.01	24,506.24 ± 2,721.44
AUC_0- ∞ _ (μg/L*h)	20,796.84 ± 3,747.31	21,546.82 ± 2,914.85	25,165.07 ± 3,018.65
C_max_ (μg/L)	1,622.2 ± 562.45	1,598.33 ± 143.02	2080 ± 399.6
T_max_ (h)	2.4 ± 0.89	4.33 ± 0.52	4.33 ± 1.37
t_1/2_ (h)	7.87 ± 4.17	6.13 ± 1.41	8.25 ± 2.87
CL_z_/F (L/h/kg)	2.96 ± 0.56	2.83 ± 0.38	2.41 ± 0.29
V_z_/F (L/kg)	35.51 ± 26.27	24.72 ± 4.85	28.28 ± 8.33
MRT_0-t_ (h)	10.12 ± 01.79	9.77 ± 1.74	9.713 ± 1.15
MRT_0- ∞ _ (h)	11.80 ± 4.35	10.14 ± 2.16	11.04 ± 2.83

Pharmacokinetic parameters are expressed as mean ± standard deviation.

### 3.3 Messenger RNA (mRNA) expression in rat liver and intestines

**FIGURE 6:**
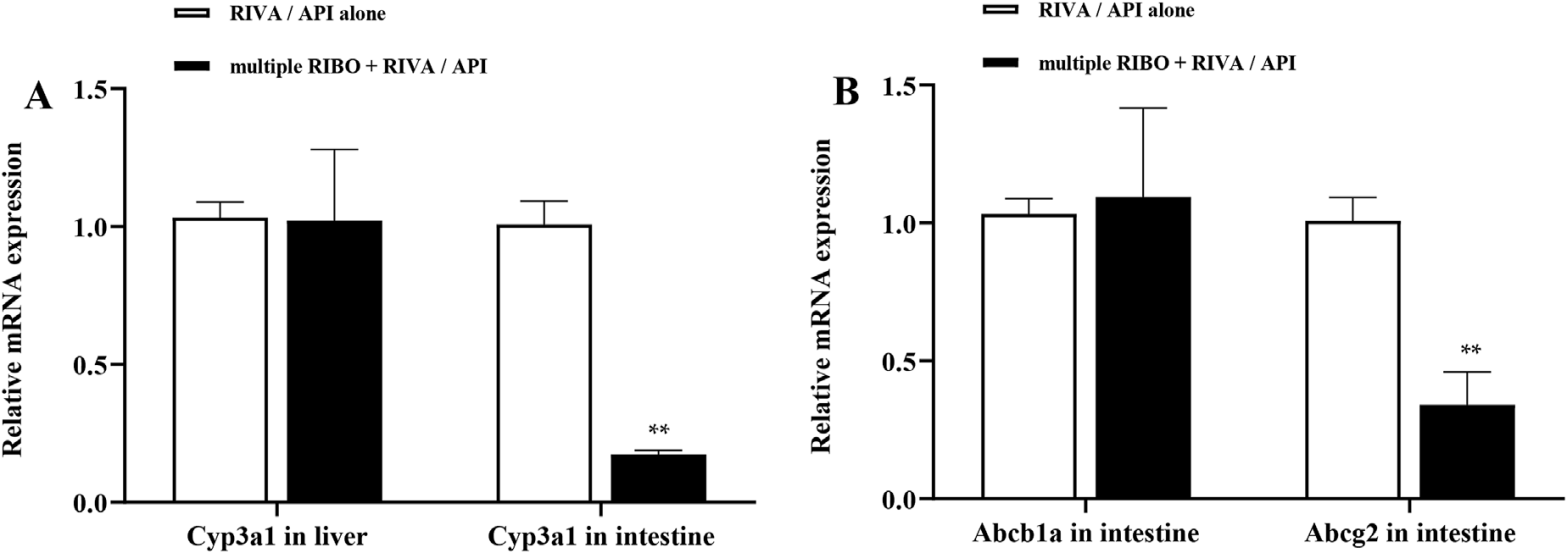
Relative mRNA expression in liver and intestine. **(A)** Effect of multiple-doses RIBO administration on mRNA expression of Cyp3a1 in liver and intestine; **(B)** Effect of multiple-doses RIBO administration on mRNA expression of Abcb1a and Abcg2 in intestine. **P < 0.01.

To investigate the possible mechanism underlying the pharmacokinetic interactions between ribociclib and rivaroxaban or apixaban involving transporters and metabolic enzymes, we assessed the mRNA expression of Abcb1a, Abcg2, and Cyp3a1 in the liver and intestines of rats. Figure 6 illustrates that continuous administration of ribociclib for 8 days significantly suppressed the mRNA expression of intestinal Cyp3a1 and Abcg2, with inhibition rates of 82.6% (P < 0.001) and 45.0% (P = 0.001), respectively. However, no significant changes were observed in the mRNA expression of Cyp3a1 in the liver or Abcb1a in the intestine.

## 4 Discussion

This study demonstrated a significant pharmacokinetic interaction between ribociclib and the oral anticoagulants rivaroxaban, while showing less effect on apixaban. The difference in results may result from the different pharmacokinetics and pharmacodynamics associated with rivaroxaban and apixaban The substantial increase in drug exposure, particularly with rivaroxaban, raises concerns about an elevated risk of bleeding in patients with cancer. This study is one of the first to examine the interaction between ribociclib and DOACs to inform the use of anticoagulants in patients with BrCAT.

Multiple doses of ribociclib combined with rivaroxaban (2 mg/kg) resulted in a significant increase in exposure to rivaroxaban, with its AUC increasing nearly 2.4-fold and its C_max_ increasing 1.9-fold compared to the control group. In addition, the CL_Z_/F of rivaroxaban decreased with the use of ribociclib. Multiple doses of ribociclib combined with apixaban (0.5 mg/kg) resulted in a 60.82% increase in the AUC of apixaban. This finding may be due to the inhibitory effect of ribociclib on metabolic enzyme,and transporters.

CYP enzymes, particularly CYP3A4, are central to the metabolism of numerous endogenous and exogenous substances. CYP3A4, expressed predominantly in the liver and the small intestine, plays a vital role in first-pass metabolism, impacting the bioavailability of many drugs. The uptake and elimination of DOACs like rivaroxaban and apixaban depend on P-gp and BCRP efflux transporter systems. Both CYP3A4 and these transporters are highly susceptible to induction or inhibition by various compounds, frequently leading to DDIs. Evidence from rat experiments with almonertinib, another inhibitor of CYP3A4, P-gp, and BCRP, showed over three fold increases in the AUC and C_max_ of rivaroxaban and apixaban (Wang et al., 2023). Similarly, drugs that strongly induce or inhibit CYP3A4 and P-gp, such as phenytoin, rifampicin, carbamazepine, and ketoconazole, have been reported to significantly interact with apixaban and rivaroxaban (Byon et al., 2019; Cohen et al., 2021; Ngo et al., 2020). Studies have demonstrated that ribociclib acts as an inhibitor of CYP3A4, P-gp, and BCRP,as well as a substrate of P-gp (Sorf et al., 2018; Samant et al., 2020). However, in our study, we only observed ribociclib to inhibit CYP3A4 and BCRP mRNA expression in rat small intestine, with no effect on P-gp in rat small intestine and CYP3A4 in rat liver. We speculate that the reasons for the different expression of CYP3A4 mRNA in the intestine and liver and the different expression of P-gp mRNA and BCRP mRNA in the intestine are as follows. *In vitro* studies demonstrated that ribociclib (molecular weight 434) exhibits a half-maximal inhibitory concentration (IC_50_) of 12.8 µM against CYP3A4 (Sorf et al., 2018). In our investigation, the C_max_ of ribociclib in rats administered 60 mg/kg reached approximately 1,500 ng/mL, which is significantly lower than its IC50 for CYP3A4 inhibition. This may explain the lack of apparent effect of ribociclib on liver CYP3A4 activity. In contrast, after oral administration of ribociclib, the drug concentration in the intestine can reach levels that inhibit the IC50 values of CYP3A4, P-gp, and BCRP (Sorf et al., 2018). However, in our study, no observable effect of ribociclib on P-gp mRNA expression in rat intestine was detected. This discrepancy may be attributed to ribociclib’s role as a substrate of P-gp, whereby its interaction with P-gp likely involves competition for substrate-binding sites rather than direct suppression of P-gp mRNA. Therefore, ribociclib may compete with rivaroxaban for P-gp binding, thereby reducing the efflux of rivaroxaban. These effects reduces the rate of rivaroxaban metabolism and excretion, resulting in a decrease in CL_Z_/F of rivaroxaban. Consequently, we speculate that the increased exposure to rivaroxaban and apixaban with ribociclib is primarily due to the inhibition of metabolic and efflux transport mechanisms.

The results of the rat experiments showed that the effect of ribociclib on exposure to rivaroxaban was significantly greater than that of apixaban. We offer several possible explanations for this observation. First, the differential roles of CYP3A4 and efflux transporters in the pharmacokinetics of these drugs may explain the variation. While fluconazole and voriconazole, moderate to strong CYP3A inhibitors, show minimal effects on apixaban pharmacokinetics, they exhibit a more pronounced impact on rivaroxaban (Rohr et al., 2022). This suggests that apixaban pharmacokinetic interaction is not dependent on the singular inhibition of CYP3A4. Furthermore, the renal clearance of rivaroxaban is highly dependent on active renal secretion via P-gp and BCRP, whereas apixaban exhibits a lower dependence on these transporters. Thus, ribociclib’s potential inhibitory effect on renal transporters may result in a greater impact on rivaroxaban exposure (Hindley et al., 2023; Bratsos, 2019). In addition, plasma protein binding was approximately 87% with apixaban and higher (approximately 95%) with rivaroxaban, suggesting that ribociclib-induced displacement of rivaroxaban from plasma proteins may lead to a greater interaction likelihood (Hindley et al., 2023). Notably, evidence indicates that inhibition of a single efflux transporter does not effectively suppress the transport of apixaban in Caco-2 cells expressing multiple transporters. However, simultaneous inhibition of P-gp and BCRP causes a more substantial reduction in apixaban efflux (Zhang et al., 2013). Intestinal efflux transport has been demonstrated to affect the absorption of rivaroxaban (Kou et al., 2021). This is also consistent with the PCR results that ribociclib inhibited intestinal BCRP expression but not intestinal P-gp. This selective inhibition may be the main reason the magnitude of the effect of ribociclib was smaller in the apixaban group than in the rivaroxaban group.

Based on these pharmacokinetic results, we investigated the effect of multiple doses of ribociclib on low-dose rivaroxaban (1 mg/kg) blood levels. The results showed that the AUC and C_max_ of rivaroxaban increased by approximately 2.3-fold and 1.3-fold, respectively. This indicates that co-administration of low-dose rivaroxaban with potent inhibitors of CYP3A4, and BCRP does not achieve the intended reduction in exposure. Notably, the AUC of rivaroxaban at 1 mg/kg was not significantly different from that at 2 mg/kg, likely due to limited absorption at the higher dose. Rivaroxaban doses exceeding 20 mg exhibit low bioavailability, suggesting that dose and exposure are not proportional at higher levels. The increased bioavailability of the lower dose, combined with ribociclib’s inhibitory effects, led to similar AUC values between the 1 mg/kg and 2 mg/kg dose groups. Therefore, when combining ribociclib and rivaroxaban in clinical practice, it is necessary to actively monitor the blood concentration of rivaroxaban. Ribociclib and rivaroxaban can be administered at appropriate intervals to avoid toxic side effects. In addition, apixaban has a better safety profile when administered concomitantly with ribociclib than rivaroxaban and can be used as the preferred dosing regimen. These findings can guide the clinical use of rivaroxaban and apixaban in combination with ribociclib.

Multiple doses of rivaroxaban or apixaban did not alter the pharmacokinetic parameters of ribociclib. Previous studies indicate that ritonavir, a strong CYP3A inhibitor, increases ribociclib AUC by 3.2-fold, whereas rifampicin, a strong CYP3A inducer, reduces ribociclib AUC by 89% (Samant et al., 2020). In contrast, rivaroxaban and apixaban were not inhibited or induced by metabolism and transport only through CYP3A4, P-gp and BCRP. Even though there was a slight increase in C_max_ in the apixaban group, probably due to competition for the same transporters and metabolizing enzymes, it was not statistically significant.

Moreover, it is essential to consider the limitations of this study. First, the observed pharmacokinetic interactions may differ between rats and humans due to species-specific differences in metabolism and transporter activity. Second, we did not use a rat model of breast cancer, and there may be variations in the metabolism and transport processes between healthy rats and those with breast cancer, potentially influencing the study outcomes. Third, the underlying mechanisms of this study are require further validation through more detailed and in-depth investigations.

## 5 Conclusion

In this study, we developed and validated a sensitive, rapid, reliable, and accurate UPLC-MS/MS method for the quantification of ribociclib in rat plasma. The method was successfully applied to the pharmacokinetic interaction study in this experiment. The experimental results showed that multiple doses of ribociclib led to increased exposure to rivaroxaban, potentially increasing the risk of hemorrhage. Furthermore, the study suggests that there may be no clinically significant drug interactions between apixaban and ribociclib. Importantly, this study provides insights that may assist in optimizing dosing regimens for patients with breast cancer-associated thrombosis, helping to adjust doses and minimize toxic side effects. Given that this experiment was conducted in rats, additional clinical validation is necessary to confirm these findings in human populations.

## Data Availability

The original contributions presented in the study are included in the article/supplementary material, further inquiries can be directed to the corresponding authors.
